# Cross‐Cohort Transcriptomic Integration Identifies IFIT2 as a Translational Diagnostic Biomarker and Functional Driver of Inflammation‐Linked Tubular Injury in Chronic Kidney Disease

**DOI:** 10.1155/humu/8282277

**Published:** 2026-05-16

**Authors:** Xiaoling Zhang, Xueyan Chen, Ya Yang, Yiyuan Zhang

**Affiliations:** ^1^ Department of Critical Care Medicine, Jinhua Hospital Affiliated to Zhejiang University, Jinhua, China; ^2^ National Clinical Research Center for Kidney Diseases, Jinling Hospital, Affiliated Hospital of Medical School, Nanjing University, Nanjing, China, nju.edu.cn; ^3^ Department of Geriatrics, The Second People’s Hospital of Huai’an / The Affiliated Huai’an Hospital of Xuzhou Medical University, Huai′an, China; ^4^ Department of Nephrology, The Affiliated Huai′an No.1 People′s Hospital, Nanjing Medical University, Huai′an, China, njmu.edu.cn

**Keywords:** biomarker, chronic kidney disease, diagnostic signature, IFIT2, inflammation, interferon signaling, transcriptomics, tubular injury

## Abstract

Chronic kidney disease (CKD) is a major global health burden characterized by progressive loss of renal function, persistent inflammation, and tubular epithelial injury, yet reproducible diagnostic biomarkers that also have functional relevance remain insufficiently defined. Here, we integrated multiple independent CKD transcriptomic cohorts from GEO (GSE32591 and GSE66494 as training datasets, with GSE180394 as an external validation dataset) using rigorous batch‐effect correction and differential expression analysis to identify consistently dysregulated genes across platforms. Shared CKD‐upregulated genes were predominantly enriched in immune and inflammatory biological processes, and module‐level analyses prioritized a core set of hub genes showing stable activation across cohorts. Based on these hubs, we constructed a composite diagnostic gene signature using a standardized Z‐score–based scoring approach, which demonstrated a robust discriminative performance in both training and external validation cohorts. Among candidate genes ranked by diagnostic performance, IFIT2 emerged as a reproducibly upregulated marker with strong diagnostic utility. Mechanistically, IFIT2 was inducible in human renal tubular epithelial cells (HK‐2 and primary RPTEC) under CKD‐relevant inflammatory (IFN‐*γ*) and profibrotic (TGF‐*β*1) stimulation. Importantly, shRNA‐mediated IFIT2 knockdown mitigated IFN‐*γ*–induced reductions in cell viability, decreased apoptosis, and attenuated the induction of tubular injury markers (KIM1 and LCN2) and inflammatory mediators (IL6 and CXCL10). Together, these results support IFIT2 as a promising candidate biomarker linking inflammation to tubular injury in CKD, providing a translational rationale for further biomarker‐guided stratification and therapeutic targeting in future studies.

## 1. Introduction

Chronic kidney disease is a progressive and irreversible condition with substantial global prevalence and is associated with increased morbidity and mortality [1, 2]. Regardless of the initiating etiology, persistent inflammation and renal tubular epithelial injury are central pathological processes driving disease progression toward end‐stage renal failure [3, 4]. Renal tubular epithelial cells actively participate in inflammatory signaling, fibrotic remodeling, and immune cell recruitment, thereby amplifying renal injury rather than serving solely as passive targets.

Accumulating evidence indicates that inflammation‐related molecular networks play a pivotal role in chronic kidney disease progression [2]. Proinflammatory cytokines and chemokines within the renal microenvironment contribute to tubular dysfunction, apoptosis, and maladaptive repair. Although transcriptomic studies have identified numerous dysregulated genes in diseased kidneys, reproducible biomarkers that are both diagnostically robust and functionally relevant remain limited [5]. Many proposed candidates lack consistency across cohorts or experimental validation, restricting their translational value.

Integrative analysis of transcriptomic data from independent cohorts provides an effective strategy to identify stable disease‐associated gene signatures. Beyond diagnostic utility, prioritizing genes that actively mediate inflammatory injury in renal tubular epithelial cells is essential for linking bioinformatic findings to biological mechanisms underlying disease progression.

Interferon‐stimulated genes are key components of inflammatory signaling pathways and have been implicated in immune‐mediated tissue injury [6]. IFIT2 has been reported to regulate cellular stress responses and immune activation in nonrenal contexts, yet its role in chronic kidney disease, particularly in renal tubular epithelial cells, remains poorly defined [7].

In this study, we first performed cross‐cohort transcriptomic integration to construct and externally validate a diagnostic gene signature for CKD. We then prioritized individual candidate genes based on diagnostic performance. Separately, we conducted targeted functional experiments in human renal tubular epithelial cells to explore the mechanistic contribution of the top prioritized gene (IFIT2) to inflammation‐linked tubular injury. This dual‐approach design allows a clearer separation between the biomarker utility of the derived signature and the biological role of IFIT2, providing both diagnostic and mechanistic insights into chronic kidney disease.

## 2. Methods

### 2.1. Transcriptomic Datasets and Data Preprocessing

Publicly available gene expression datasets related to chronic kidney disease were retrieved from the Gene Expression Omnibus (GEO) database [8]. The training cohorts included GSE32591 and GSE66494, whereas GSE180394 was used as an independent validation cohort. These datasets were generated on different microarray platforms and derived from independent patient cohorts.

Raw or preprocessed expression matrices and corresponding platform annotation files were obtained. Probe identifiers were mapped to official gene symbols, and when multiple probes corresponded to the same gene, expression values were aggregated by calculating the mean. Genes without valid annotations were excluded, and only genes shared across all datasets were retained for downstream integrative analyses.

### 2.2. Batch Effect Correction and Differential Expression Analysis

**Figure 1:**
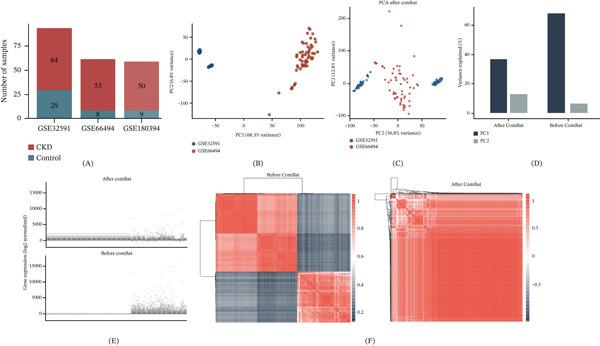
Overview of multicohort dataset integration and batch effect correction. (A) Summary of sample composition across the three CKD transcriptomic cohorts. (B) Principal component analysis (PCA) of the uncorrected combined expression matrix, revealing clear dataset‐driven separation. (C) PCA after ComBat batch correction, showing improved mixing of samples from different cohorts. (D) Proportion of variance explained by the first two principal components before and after batch correction, demonstrating reduction in technical variance. (E) Density distributions of gene expression values before and after correction. (F) Sample‐to‐sample correlation heat maps before (upper panel) and after (lower panel) correction, illustrating the removal of dataset‐specific patterns.

To reduce technical variability introduced by different microarray platforms, the combined expression matrix was corrected for batch effects using the ComBat algorithm from the sva package, with dataset origin defined as the batch variable [9, 10]. The correction process was evaluated using principal component analysis (PCA), gene expression density distributions, and sample‐to‐sample correlation heat maps, with results presented in the corresponding figures (see Results Section 3.1 and Figure 1). Differential expression analysis was subsequently performed separately in the two training cohorts using the limma package [11]. Samples were classified as chronic kidney disease or control based on curated annotations. Linear models with empirical Bayes moderation were applied, and genes with an absolute log2 fold change greater than 1 and a false discovery rate below 0.05 were considered significantly differentially expressed.

### 2.3. Identification of Shared Gene Modules and Hub Genes

Shared differentially expressed genes were defined as genes meeting differential expression criteria in both training datasets with consistent directionality. Functional enrichment analysis of shared gene sets was conducted using gene ontology biological process annotations with false discovery rate adjustment [12].

Shared upregulated genes were further used for module‐level analyses. Gene expression values were Z‐score normalized, and unsupervised hierarchical clustering was performed using Euclidean distance and complete linkage. Hub genes were selected based on robust differential expression between chronic kidney disease and control samples and consistent activation across cohorts. For each sample, a hub gene module score was calculated as the mean Z‐score expression of the selected hub genes and compared between groups using nonparametric statistical tests.

### 2.4. Construction and Evaluation of the Diagnostic Gene Signature

Model genes were selected from the identified hub genes to construct a composite diagnostic gene signature. Gene expression values were Z‐score normalized within each cohort, and a signature score was calculated for each sample as the average Z‐score of all selected model genes.

This straightforward averaging approach was chosen for its simplicity and high reproducibility. Diagnostic performance was assessed by receiver operating characteristic (ROC) curve analysis with corresponding area under the curve (AUC) values [13]. The consistency and robustness of the signature were further evaluated in an independent external validation cohort (GSE180394) using the identical scoring procedure.

### 2.5. Cell Culture

Human renal proximal tubular epithelial cells, including the HK‐2 cell line (ATCC, RRID: CVCL_0302) and primary RPTEC cells (ATCC, RRID: CVCL_K278), were used in this study. HK‐2 cells were cultured in DMEM/F12 supplemented with 10% fetal bovine serum, whereas RPTEC cells were maintained in Renal Epithelial Cell Basal Medium with growth supplements, according to the manufacturer′s instructions. All cells were cultured at 37°C in a humidified atmosphere containing 5% CO_2_ and used within 10 passages. Cells were routinely tested and confirmed to be free of mycoplasma contamination.

### 2.6. Cell Treatment and CKD‐Related Stimulation

To mimic chronic kidney disease–associated inflammatory and profibrotic conditions, renal tubular epithelial cells were stimulated with recombinant human interferon‐*γ* (IFN‐*γ*, 10 ng/mL) or transforming growth factor‐*β*1 (TGF‐*β*1, 5 ng/mL) for the indicated time points (0, 8, 24, and 48 h). For gene knockdown experiments, cells were transduced with shRNA targeting IFIT2 or nontargeting control shRNA. After 36–48 h of transduction, cells were subjected to IFN‐*γ* stimulation. Untreated cells and shRNA control–transduced cells served as controls. Cells were subsequently harvested for RNA analysis, cell viability assays, or flow cytometric analysis, as appropriate.

### 2.7. RNA Extraction, Reverse Transcription, and Quantitative Real‐Time PCR

Total RNA was extracted using TRIzol reagent, and RNA concentration and purity were assessed by spectrophotometry. Equal amounts of RNA (1 *μ*g) were reverse‐transcribed into cDNA using a commercial reverse transcription kit. Quantitative real‐time PCR was performed using SYBR Green chemistry under standard cycling conditions. GAPDH was used as an internal reference, and relative gene expression was calculated using the 2^−*Δ* *Δ* *C* *t*^ method. All experiments were conducted in triplicate. Primer sequences used in this study are listed below:

• GAPDH‐F: TTCTTTTGCGTCGCCAGCC
• GAPDH‐R: TCCCGTTCTCAGCCTTGAC
• IFIT2‐F: GGAACCTGGTGACTAAGGGC
• IFIT2‐R: AACCCAGAGTGTGGCTGATG
• KIM1‐F: TCCGTGGCCCTTTTTGCTTA
• KIM1‐R: CTGCCTCTCCACCAACCTTT
• LCN2‐F: GGGAGAACCAAGGAGCTGAC
• LCN2‐R: CGGCACCTGTGCACTCA
• IL6‐F: CCACCGGGAACGAAAGAGAA
• IL6‐R: GAGAAGGCAACTGGACCGAA
• CXCL10‐F: GTGGATGTTCTGACCCTGCT
• CXCL10‐R: GGAGGATGGCAGTGGAAGTC

### 2.8. Cell Viability Assay (CCK‐8)

Cell viability was assessed using the Cell Counting Kit‐8 assay. HK‐2 and RPTEC cells were seeded into 96‐well plates, transduced with shRNA targeting IFIT2 or control shRNA, and stimulated with IFN‐*γ* as described above. At the indicated time points (0, 24, 48, 72, and 96 h), CCK‐8 reagent was added, and absorbance was measured at 450 nm. Cell viability was expressed as a percentage relative to the corresponding control group. Experiments were performed with three technical replicates and independently repeated at least three times.

### 2.9. Flow Cytometry Analysis of Apoptosis

Apoptosis was analyzed using Annexin V‐FITC/propidium iodide staining followed by flow cytometry. After shRNA transduction and IFN‐*γ* stimulation for 48 h, cells were harvested, stained according to the manufacturer′s instructions, and analyzed using a flow cytometer. Data were collected from at least 10,000 cells per sample and analyzed using the FlowJo software. Early and late apoptotic cells were quantified as percentages of total cells. All experiments were independently repeated at least three times.

### 2.10. Statistical Analysis

Data are presented as mean ± standard deviation. Statistical analyses were performed using the GraphPad Prism software. Comparisons between two groups were conducted using unpaired two‐tailed Student′s *t* tests, whereas multiple‐group comparisons were performed using one‐way ANOVA followed by Tukey′s post hoc test. A *p* value of < 0.05 was considered statistically significant.

## 3. Results

### 3.1. Integration of Multicohort Transcriptomic Data and Batch Effect Correction

To enable cross‐cohort integrative analysis, three independent microarray datasets generated on different platforms were combined (Figure 1A). PCA of the uncorrected expression matrix showed clear separation of samples by dataset origin, confirming the presence of substantial batch effects (Figure 1B). Following ComBat correction, samples from different cohorts exhibited improved intermixing, with a noticeable reduction in dataset‐associated variance along the leading principal components (Figure 1C–D). These improvements were further supported by more uniform gene expression density distributions and enhanced sample‐to‐sample correlations after correction (Figure 1E–F). Overall, batch effects were successfully mitigated, producing a comparable expression matrix suitable for downstream differential expression and signature analyses.

### 3.2. Identification of Reproducible Differentially Expressed Genes Across CKD Transcriptomic Cohorts

**Figure 2:**
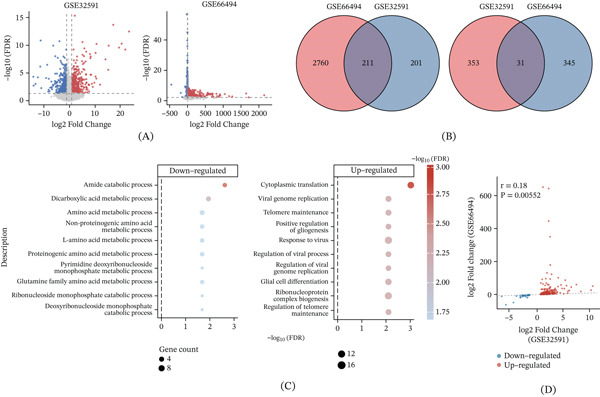
Identification and functional characterization of shared differentially expressed genes in CKD. (A) Volcano plots showing differentially expressed genes between CKD and control samples in the training datasets GSE32591 and GSE66494 after batch correction. (B) Venn diagrams illustrating the overlap of significantly upregulated (left) and downregulated (right) genes between the two cohorts. (C) Gene ontology biological process enrichment analysis of shared upregulated and downregulated genes, visualized as dot plots based on −log10(FDR) and gene counts. (D) Scatter plot showing the correlation of log2 fold changes for shared differentially expressed genes between GSE32591 and GSE66494.

Differential expression analysis was performed independently in the two training cohorts after batch correction (Figure 2A). A large number of genes were differentially expressed in both datasets, with a markedly asymmetric overlap pattern: 211 genes were consistently upregulated, whereas only 31 genes showed concordant downregulation (Figure 2B). Functional enrichment analysis revealed that shared upregulated genes were predominantly associated with immune activation, inflammatory responses, and fibrotic processes, whereas shared downregulated genes showed limited enrichment (Figure 2C). Cross‐cohort comparison of fold changes demonstrated a modest but significant positive correlation, with greater concordance observed among upregulated genes (Figure 2D), supporting the robustness of inflammation‐related transcriptional signatures in CKD.

### 3.3. Identification of Core Pathogenic Modules and Hub Genes in CKD

Unsupervised clustering based on shared upregulated genes revealed consistent activation patterns across CKD samples from both training cohorts, with clear separation from control samples (Figure S1A). Within this gene set, a subset of hub genes exhibited robust and coordinated upregulation in CKD across cohorts (Figure S1B). A module score calculated from these hub genes was significantly higher in CKD samples compared with controls in both datasets (Figure S1C), indicating a stable disease‐associated transcriptional module.

### 3.4. Construction and Validation of a CKD Diagnostic Gene Signature

**Figure 3:**
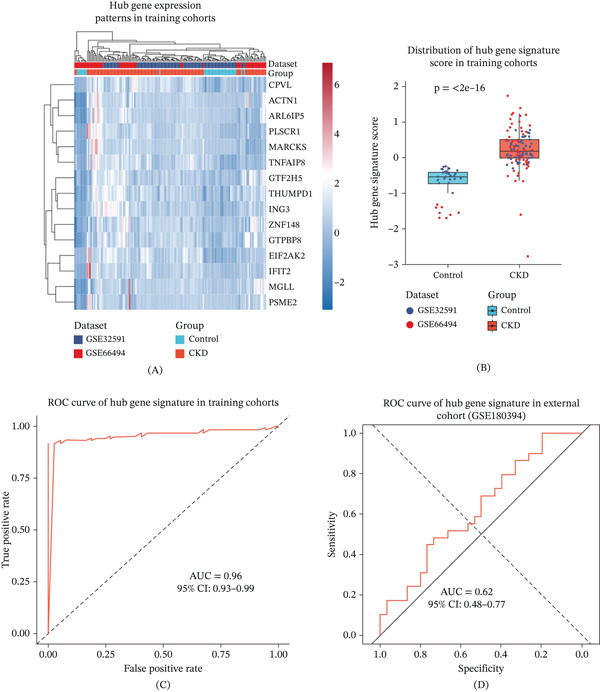
Construction and evaluation of a CKD diagnostic gene signature. (A) Heat map showing the expression patterns of model genes in the training cohorts. Gene expression values were Z‐score normalized, and samples were annotated by disease status and dataset. (B) Distribution of the gene signature score between control and CKD samples in the training cohorts. Each dot represents one sample, and statistical significance was assessed between groups. (C) Receiver operating characteristic (ROC) curve evaluating the diagnostic performance of the gene signature in the training cohorts. (D) ROC curve demonstrating the diagnostic performance of the gene signature in an independent external validation cohort (GSE180394).

Based on the identified hub genes, a diagnostic gene signature for chronic kidney disease was constructed and evaluated. In the training cohorts, expression patterns of model genes clearly distinguished CKD samples from controls (Figure 3A), and the composite signature score was significantly elevated in CKD samples (Figure 3B).

ROC analysis demonstrated encouraging diagnostic performance with high AUC values in the training cohorts (Figure 3C). This performance was independently validated in an external cohort (GSE180394), where the signature maintained good discriminative capacity (Figure 3D). The consistent performance across training and validation datasets supports the stability of the Z‐score–based signature under the applied analytical framework.

### 3.5. Characterization and Prioritization of Model Genes in CKD

**Figure 4:**
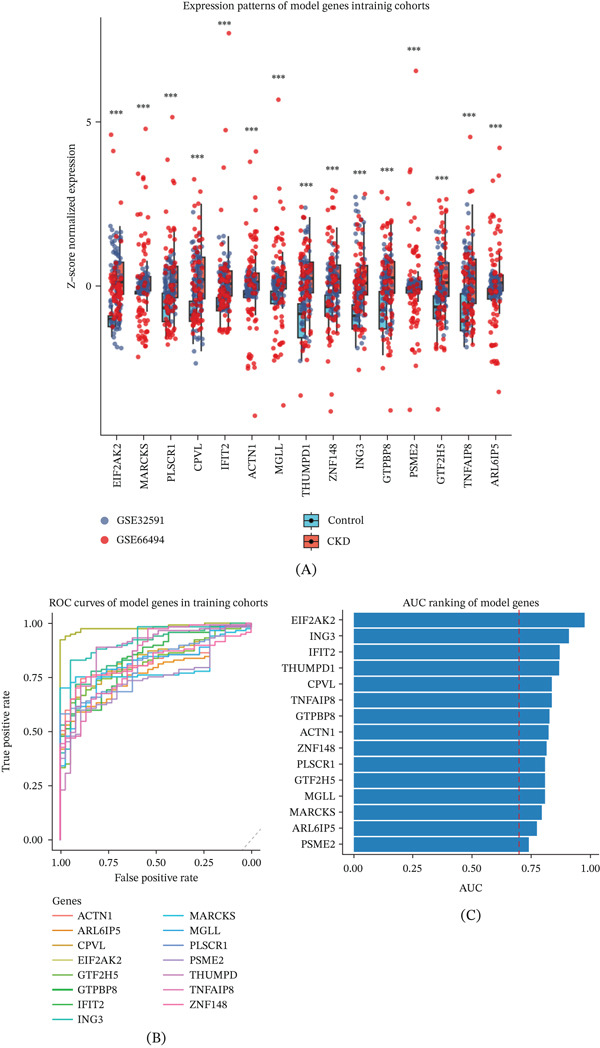
Prioritization and diagnostic performance of model genes in CKD. (A) Differential expression patterns of model genes between CKD and control samples in the training cohorts. Gene expression values were Z‐score normalized across samples. Boxplots represent the median and interquartile range, with individual points indicating each sample. Statistical significance between CKD and control groups was assessed using the Wilcoxon rank‐sum test, and significance levels are indicated above each gene (*p* < 0.05; *p* < 0.01; and *p* < 0.001). (B) Receiver operating characteristic (ROC) curves of individual model genes for distinguishing CKD from control samples in the training cohorts. Each curve represents the diagnostic performance of a single gene. (C) Area under the ROC curve (AUC) ranking of model genes. Genes are ordered according to their AUC values, and the dashed red line indicates an AUC of 0.70, highlighting genes with relatively strong diagnostic potential.

To characterize individual components of the diagnostic gene signature, we examined the expression patterns and diagnostic performance of each model gene in the training cohorts. All model genes exhibited significant differential expression between CKD and control samples and showed consistent expression trends across datasets (Figure 4A). ROC curve analysis demonstrated variable discriminative ability among individual genes (Figure 4B). The ranking by AUC identified EIF2AK2, ING3, IFIT2, and THUMPD1 as the top‐performing candidates, each achieving an AUC greater than 0.80 (Figure 4C).

Although EIF2AK2, ING3, and THUMPD1 also displayed strong diagnostic potential, IFIT2 was prioritized for subsequent functional validation based on its consistent cross‐cohort upregulation, established involvement in interferon signaling pathways, and biological plausibility in inflammation‐associated tubular injury. This prioritization reflects a focused approach to link computational ranking with targeted mechanistic experiments rather than implying that the top candidates are entirely interchangeable or that other genes lack potential value.

### 3.6. IFIT2 Is Induced by Inflammatory and Fibrotic Stimuli in Renal Tubular Epithelial Cells

**Figure 5:**
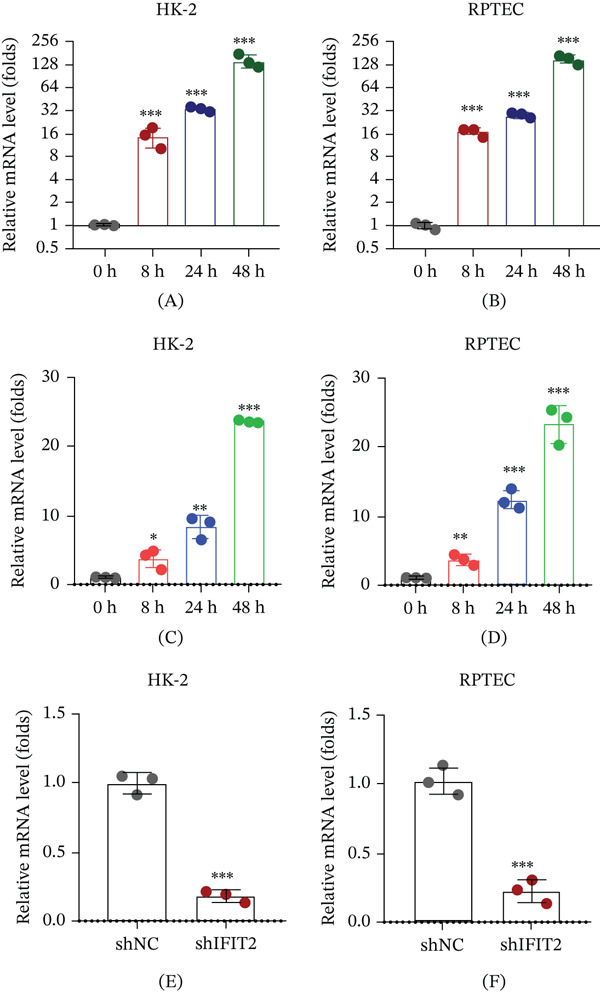
Induction and knockdown of IFIT2 in renal tubular epithelial cells. (A–B) IFN‐*γ*–induced IFIT2 expression in HK‐2 and RPTEC cells. (C–D) TGF‐*β*1–induced IFIT2 expression in HK‐2 and RPTEC cells. (E–F) Validation of IFIT2 knockdown efficiency by qPCR. Data are presented as mean ± SD. ∗*p* < 0.05, ∗∗*p* < 0.01, and ∗∗∗*p* < 0.001.

Renal tubular epithelial cells were exposed to inflammatory and profibrotic stimuli to assess whether IFIT2 responds to pathological conditions relevant to chronic kidney disease. IFN‐*γ* stimulation induced a significant and time‐dependent upregulation of IFIT2 mRNA expression in both HK‐2 and RPTEC cells, with marked increases observed at 24 h and further elevation at 48 h (Figure 5A–B). Similarly, treatment with TGF‐*β*1 significantly increased IFIT2 expression in both cell types (Figure 5C–D). For subsequent functional analyses, IFIT2 was efficiently silenced using shRNA in HK‐2 and RPTEC cells, resulting in a pronounced reduction of IFIT2 mRNA levels compared with control cells (Figure 5E–F). These results demonstrate that IFIT2 is inducible under inflammatory and fibrotic conditions associated with chronic kidney disease.

### 3.7. IFIT2 Knockdown Attenuates IFN‐*γ*–Induced Injury and Apoptosis in Renal Tubular Epithelial Cells

**Figure 6:**
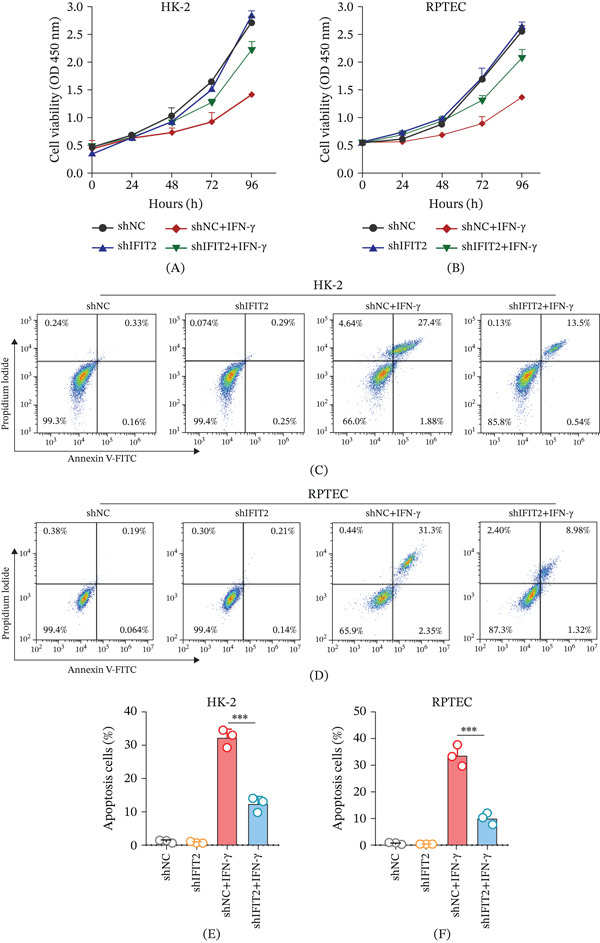
IFIT2 knockdown attenuates IFN‐*γ*–induced injury and apoptosis in renal tubular epithelial cells. (A–B) CCK‐8 assay showing that IFIT2 knockdown alleviates IFN‐*γ*–induced reduction of cell viability in HK‐2 and RPTEC cells. (C–F) Annexin V/PI flow cytometry analysis showing that IFIT2 knockdown reduces IFN‐*γ*–induced apoptosis in (C, E) HK‐2 and (D, F) RPTEC cells. Data are presented as mean ± SD from three independent experiments. ∗∗∗*p* < 0.001.

To determine whether IFIT2 functionally contributes to inflammatory injury in renal tubular epithelial cells, the effects of IFIT2 knockdown on IFN‐*γ*–induced changes in cell viability and apoptosis were evaluated. IFN‐*γ* exposure resulted in a progressive decline in cell viability in both HK‐2 and RPTEC cells over time, as measured by the CCK‐8 assay (Figure 6A–B). In contrast, silencing IFIT2 significantly mitigated the IFN‐*γ*–induced reduction in cell viability, with shIFIT2‐transduced cells maintaining higher viability compared with shNC controls under inflammatory conditions. Consistent with these findings, IFN‐*γ* stimulation markedly increased apoptotic cell populations in renal tubular epithelial cells, whereas IFIT2 knockdown substantially reduced IFN‐*γ*–induced apoptosis in both HK‐2 (Figure 6C,E) and RPTEC cells (Figure 6D,F). Collectively, these results indicate that IFIT2 plays a functional role in mediating IFN‐*γ*–induced inflammatory injury and apoptotic responses in renal tubular epithelial cells, and that suppression of IFIT2 can partially reverse CKD‐associated cellular abnormalities.

### 3.8. IFIT2 Knockdown Attenuates IFN‐*γ*–Induced Expression of Renal Tubular Injury Markers

To further evaluate the role of IFIT2 in renal tubular injury under inflammatory conditions, the expression of established tubular injury markers was examined following IFIT2 knockdown and IFN‐*γ* stimulation. IFN‐*γ* treatment markedly increased the mRNA expression levels of KIM1 and LCN2 in both HK‐2 and RPTEC cells, indicating pronounced tubular injury responses. In contrast, silencing IFIT2 significantly attenuated IFN‐*γ*–induced upregulation of KIM1 and LCN2 in HK‐2 cells (Figure S2A–B) as well as in RPTEC cells (Figure S2C–D). These findings suggest that IFIT2 contributes to IFN‐*γ*–mediated tubular injury signaling and that suppression of IFIT2 alleviates inflammatory stress–induced renal tubular damage at the molecular level.

### 3.9. IFIT2 Knockdown Attenuates IFN‐*γ*–Induced Inflammatory and Chemokine Responses in Renal Tubular Epithelial Cells

To investigate whether IFIT2 participates in IFN‐*γ*–driven inflammatory signaling in renal tubular epithelial cells, the expression of proinflammatory cytokines and chemokines was examined following IFIT2 knockdown. IFN‐*γ* stimulation markedly increased the mRNA expression levels of IL6 and CXCL10 in both HK‐2 and RPTEC cells, reflecting an activated inflammatory and chemokine response. In contrast, silencing IFIT2 significantly attenuated IFN‐*γ*–induced upregulation of IL6 and CXCL10 in HK‐2 cells (Figure S3A–B) as well as in RPTEC cells (Figure S3C–D). These findings indicate that IFIT2 contributes to IFN‐*γ*–mediated inflammatory and chemokine signaling in renal tubular epithelial cells, and that suppression of IFIT2 alleviates inflammation‐associated cellular responses relevant to CKD.

## 4. Discussion

Chronic kidney disease is characterized by persistent inflammation and progressive tubular injury, with renal tubular epithelial cells acting as active contributors to inflammatory amplification rather than passive targets [14, 15]. Elucidating molecular mediators that connect inflammatory signaling to tubular cell dysfunction is therefore essential for identifying clinically relevant biomarkers and therapeutic targets.

In this study, we integrated transcriptomic data from multiple independent chronic kidney disease cohorts to construct a robust diagnostic gene signature and to prioritize biologically relevant candidate genes. Among the model genes, IFIT2 was prioritized for functional validation due to its strong diagnostic ranking (AUC> 0.80), consistent upregulation across cohorts, and known role in interferon‐driven responses. This selection complements rather than replaces the potential utility of other top‐ranked candidates such as EIF2AK2, ING3, and THUMPD1 [16].

IFIT2 belongs to the interferon‐stimulated gene family and has been implicated in immune regulation and cellular stress responses in diverse disease contexts [6, 17]. Interferon‐driven pathways are increasingly recognized as contributors to renal inflammation; however, the specific role of this gene in chronic kidney disease, particularly within renal tubular epithelial cells, has not been well‐defined [18, 19]. Our data demonstrate that IFIT2 is inducible by both inflammatory and profibrotic stimuli relevant to chronic kidney disease, supporting its involvement at the interface of immune activation and tubular injury.

Functional analyses further showed that IFIT2 knockdown attenuated IFN‐*γ*–induced loss of cell viability and apoptosis in renal tubular epithelial cells, indicating a contributory role in inflammation‐associated cellular injury [20, 21]. As a classic interferon‐stimulated gene, IFIT2 likely participates in amplifying intracellular inflammatory signaling cascades triggered by IFN‐*γ*, thereby contributing to the maladaptive stress responses of tubular epithelial cells under persistent inflammatory conditions in CKD [6, 17].

Subsequent shRNA‐mediated knockdown of IFIT2 significantly attenuated IFN‐*γ*–induced reductions in cell viability, decreased the proportion of apoptotic cells, and suppressed the upregulation of established tubular injury markers (KIM1 and LCN2) as well as proinflammatory mediators (IL6 and CXCL10) (Figures 6, S2, and S3). These coordinated phenotypic rescues indicate that IFIT2 functions as an important node linking interferon‐driven signaling to downstream tubular injury and inflammatory amplification programs within renal epithelial cells. By mitigating these responses upon knockdown, IFIT2 suppression appears to partially restore cellular homeostasis under inflammatory stress relevant to CKD progression.

Although these loss‐of‐function experiments provide clear evidence of IFIT2′s biological relevance and contributory role at the phenotypic level, we acknowledge that causal inference would be further strengthened by complementary validation strategies, such as rescue experiments or gain‐of‐function approaches. Such experiments will be valuable to more firmly anchor the mechanistic conclusions in future studies. In addition to effects on cell survival, suppression of IFIT2 reduced the expression of established tubular injury markers and inflammatory mediators. Kidney injury molecule 1 and lipocalin 2 are widely used indicators of tubular damage, whereas interleukin 6 and C‐X‐C motif chemokine ligand 10 are key drivers of immune cell recruitment and sustained inflammatory signaling [22–25]. The coordinated attenuation of these markers suggests that IFIT2 may act upstream in regulating inflammatory amplification within tubular epithelial cells.

From a clinical perspective, diagnostic biomarkers for chronic kidney disease are often limited by insufficient specificity or lack of mechanistic relevance [26, 27]. The gene signature and IFIT2 showed encouraging performance within the analyzed curated transcriptomic cohorts. However, their generalizability to real‐world heterogeneous clinical samples—such as those with varying tissue quality, comorbid inflammatory conditions, or mixed etiologies—remains to be fully evaluated in larger, more diverse patient cohorts. Future validation in such heterogeneous settings will be essential to better assess the translational potential of IFIT2 as a biomarker component. Modulating inflammatory pathways in tubular epithelial cells may therefore represent a strategy to mitigate renal injury and disease progression [28, 29].

Several limitations should be acknowledged. The functional role of IFIT2 was validated in vitro using human renal tubular epithelial cells, and in vivo validation was not performed. Moreover, the downstream signaling mechanisms through which IFIT2 modulates inflammatory and injury responses remain to be elucidated. Future studies incorporating animal models or human renal tissue samples will be necessary to further define its role in the complex renal microenvironment.

## 5. Conclusion

This study identifies a reproducible diagnostic gene signature for CKD through a multicohort transcriptomic integration and highlights IFIT2 as a promising candidate biomarker with encouraging diagnostic performance. Complementary functional experiments position IFIT2 as a mediator linking interferon‐driven inflammation to tubular epithelial injury in renal cells. By clearly separating the biomarker utility from the mechanistic insights, our findings provide a translational foundation for further evaluation of IFIT2 in biomarker‐guided stratification and for exploring interferon‐related pathways as potential therapeutic targets in chronic kidney disease.

## Author Contributions

X.Z., X.C., and Y.Y. conceived and designed the study. X.Z. and X.C. performed data acquisition, bioinformatic analyses, and visualization. Y.Z. assisted with clinical interpretation and data curation. X.Z. and X.C. conducted the in vitro experiments and collected experimental data. Y.Y. and Y.Z. supervised the project and provided critical revisions. X.Z. drafted the manuscript. X.Z. and X.C. have contributed equally to this work and share first authorship.

## Funding

This study was supported by the Zhejiang Provincial TCM Sci‐Tech Program (No. 2023ZL188).

## Disclosure

All authors contributed to manuscript revision, read, and approved the final version.

## Conflicts of Interest

The authors declare no conflicts of interest.

## Supporting Information

Additional supporting information can be found online in the Supporting Information section.

## Supporting information


**Supporting Information 1** Figure S1: Identification of core pathogenic modules and hub genes. (A) Heat map showing the expression patterns of shared upregulated genes across the training cohorts (GSE32591 and GSE66494). Gene expression values were Z‐score normalized by gene, and samples were annotated by disease status (CKD vs. control) and dataset origin. (B) Expression profiles of selected hub genes across CKD and control samples in the training cohorts. Each point represents an individual sample, and statistical significance was assessed using two‐sided Wilcoxon rank‐sum tests. (C) Distribution of hub gene module scores in CKD and control samples across training cohorts. Module scores were calculated as the mean Z‐score expression of hub genes per sample, highlighting consistent elevation of module activity in CKD.

**Supporting Information 2** Figure S2: IFIT2 knockdown attenuates IFN‐*γ*–induced expression of renal tubular injury markers. (A) KIM1 expression in HK‐2 cells. (B) LCN2 expression in HK‐2 cells. (C) KIM1 expression in RPTEC cells. (D) LCN2 expression in RPTEC cells. Expression levels were normalized to GAPDH and presented as fold changes relative to the shNC group. Data are shown as mean ± SD from three independent experiments. ∗*p* < 0.05, ∗∗*p* < 0.01, and ∗∗∗*p* < 0.001.

**Supporting Information 3** Figure S3: IFIT2 knockdown attenuates IFN‐*γ*–induced inflammatory and chemokine responses in renal tubular epithelial cells. (A) IL6 expression in HK‐2 cells. (B) CXCL10 expression in HK‐2 cells. (C) IL6 expression in RPTEC cells. (D) CXCL10 expression in RPTEC cells. Expression levels were normalized to GAPDH and presented as fold changes relative to the shNC group. Data are shown as mean ± SD from three independent experiments. ∗*p* < 0.05, ∗∗*p* < 0.01, and ∗∗∗*p* < 0.001.

## Data Availability

The transcriptomic datasets analyzed in this study are publicly available in the Gene Expression Omnibus (GEO) repository. Additional data supporting the findings of this study are available from the corresponding authors upon reasonable request.
