# Supplementary figures and images for: Cross‐Cohort Transcriptomic Integration Identifies IFIT2 as a Translational Diagnostic Biomarker and Functional Driver of Inflammation‐Linked Tubular Injury in Chronic Kidney Disease
Source: Hum Mutat. 2026 May 16;2026:8282277. doi: 10.1155/humu/8282277 (PMC13179815; doi:10.1155/humu/8282277)

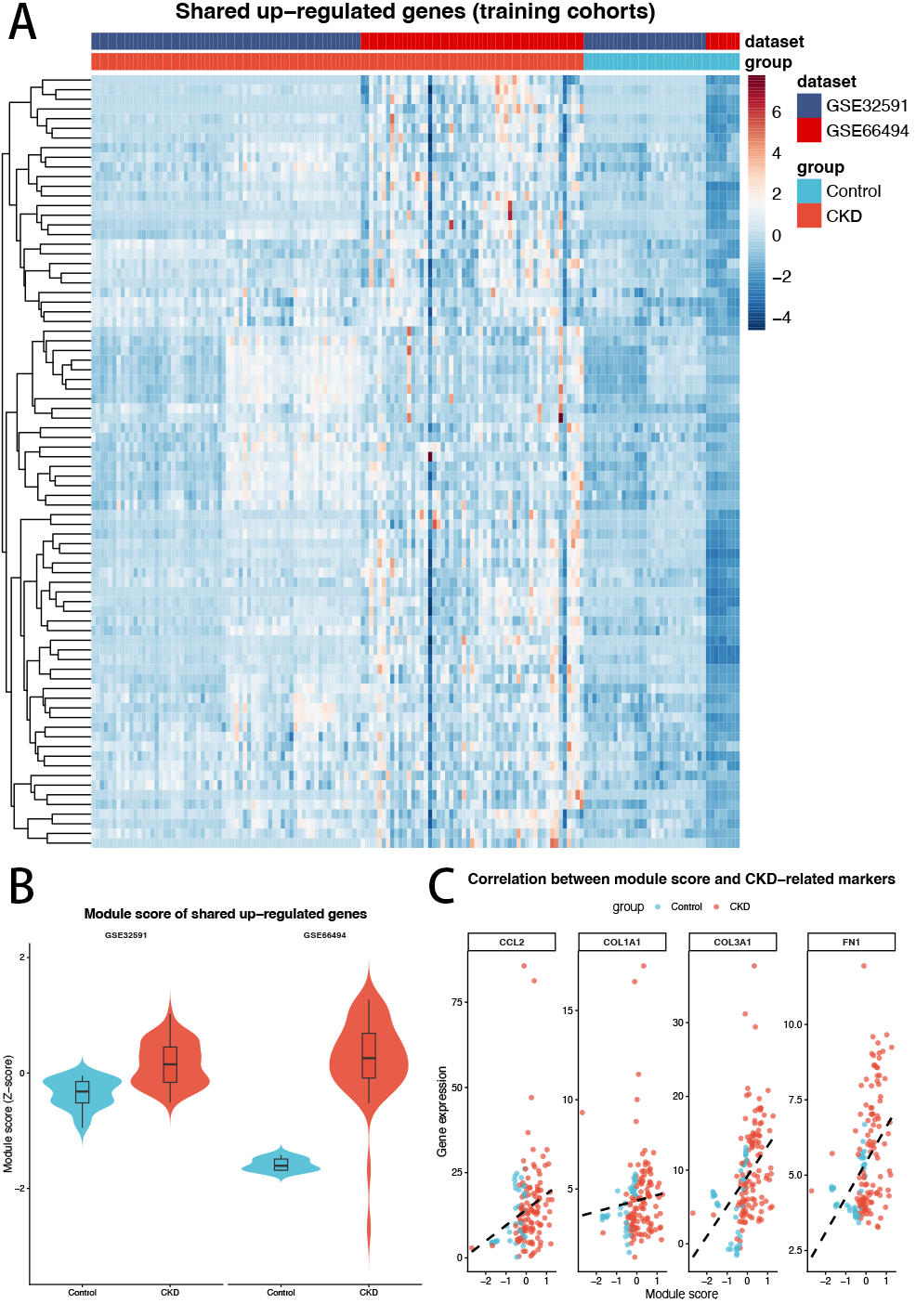

Supplement: Supplementary file 1 — Supporting Information 1 Figure S1: Identification of core pathogenic modules and hub genes. (A) Heat map showing the expression patterns of shared upregulated genes across the training cohorts (GSE32591 and GSE66494). Gene expression values were Z‐score normalized by gene, and samples were annotated by disease status (CKD vs. control) and dataset origin. (B) Expression profiles of selected hub genes across CKD and control samples in the training cohorts. Each point represents an individual sample, and statistical significance was assessed using two‐sided Wilcoxon rank‐sum tests. (C) Distribution of hub gene module scores in CKD and control samples across training cohorts. Module scores were calculated as the mean Z‐score expression of hub genes per sample, highlighting consistent elevation of module activity in CKD. [file HUMU-2026-8282277-s001.tif]

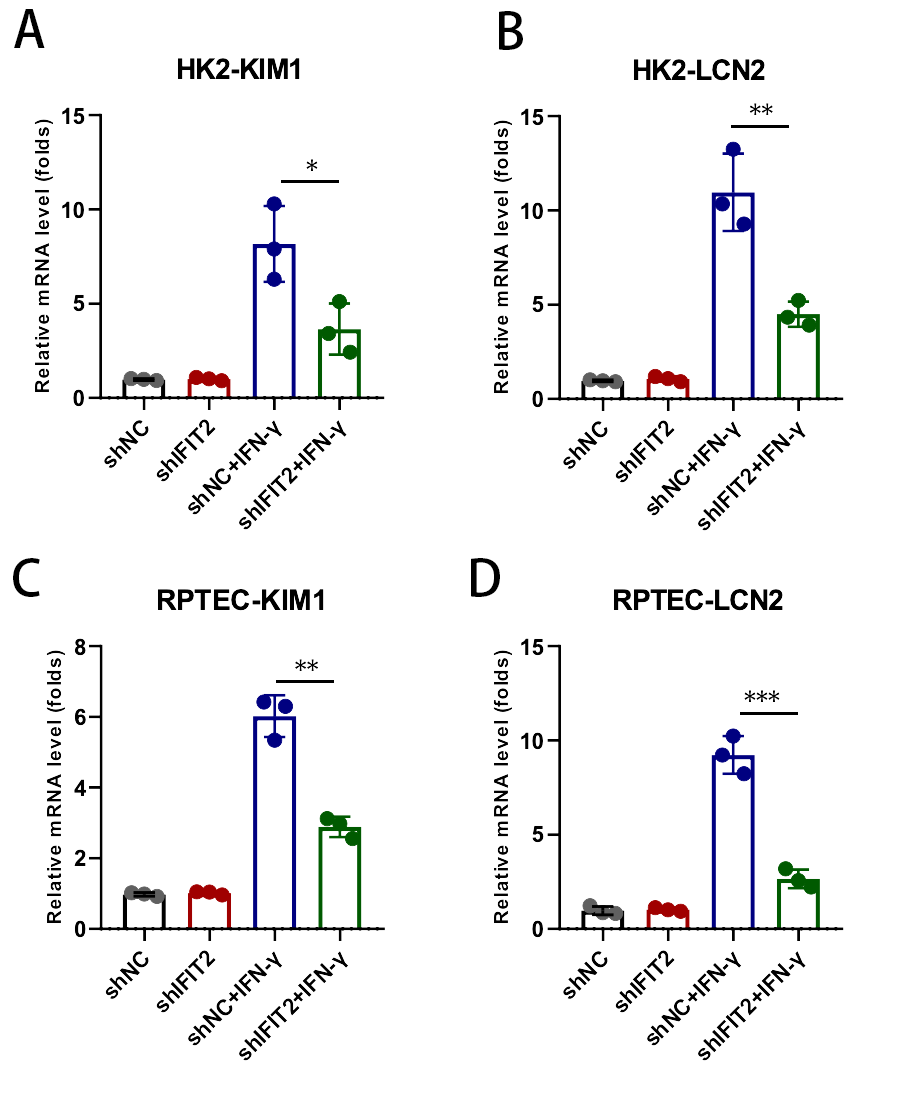

Supplement: Supplementary file 2 — Supporting Information 2 Figure S2: IFIT2 knockdown attenuates IFN‐γ–induced expression of renal tubular injury markers. (A) KIM1 expression in HK‐2 cells. (B) LCN2 expression in HK‐2 cells. (C) KIM1 expression in RPTEC cells. (D) LCN2 expression in RPTEC cells. Expression levels were normalized to GAPDH and presented as fold changes relative to the shNC group. Data are shown as mean ± SD from three independent experiments. ∗p < 0.05, ∗∗p < 0.01, and ∗∗∗p < 0.001. [file HUMU-2026-8282277-s002.tif]

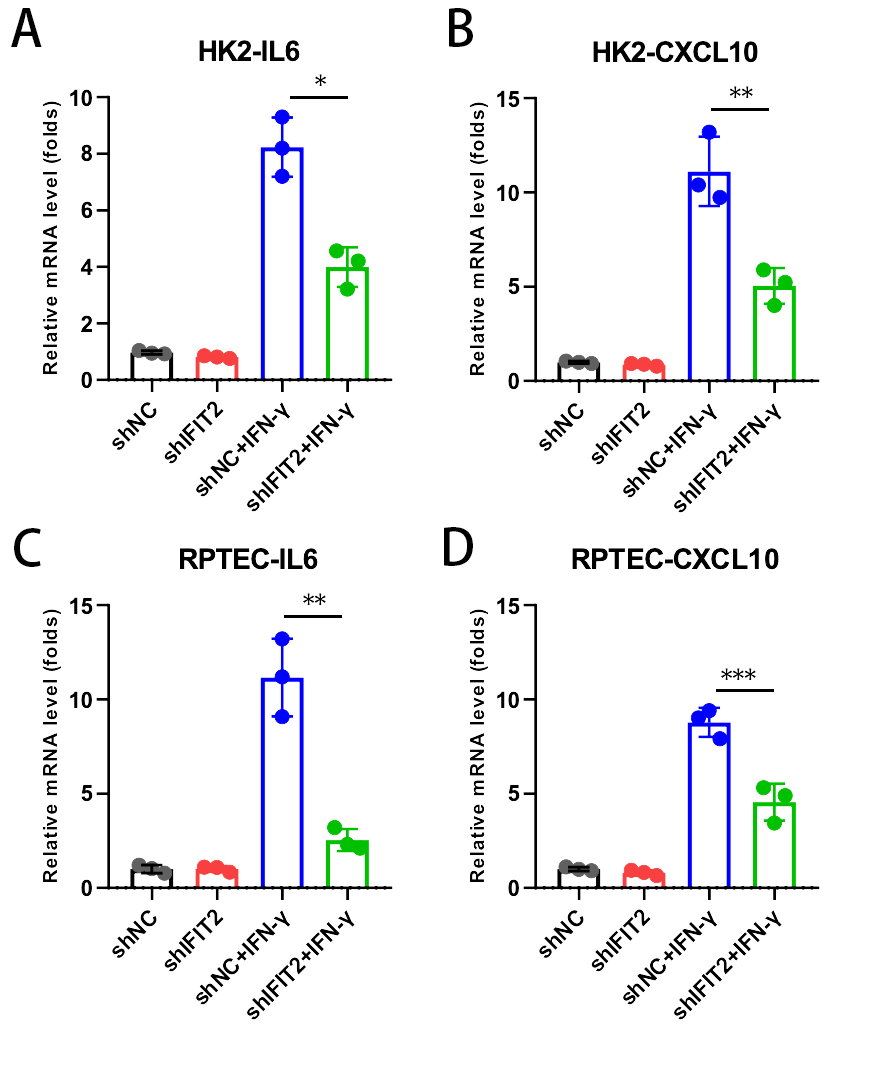

Supplement: Supplementary file 3 — Supporting Information 3 Figure S3: IFIT2 knockdown attenuates IFN‐γ–induced inflammatory and chemokine responses in renal tubular epithelial cells. (A) IL6 expression in HK‐2 cells. (B) CXCL10 expression in HK‐2 cells. (C) IL6 expression in RPTEC cells. (D) CXCL10 expression in RPTEC cells. Expression levels were normalized to GAPDH and presented as fold changes relative to the shNC group. Data are shown as mean ± SD from three independent experiments. ∗p < 0.05, ∗∗p < 0.01, and ∗∗∗p < 0.001. [file HUMU-2026-8282277-s003.tif]
